# Elastic tubes: the ideal equipment for telehealth exercise medicine in the management of prostate cancer?

**DOI:** 10.1007/s00520-022-06858-1

**Published:** 2022-02-01

**Authors:** Georgios Mavropalias

**Affiliations:** 1 Exercise Medicine Research Institute, Edith Cowan University, 270 Joondalup Drive, Joondalup, WA 6027 Australia; 2 School of Medical and Health Sciences, Edith Cowan University, Joondalup, Australia

**Keywords:** Exercise medicine, Telehealth, Home-based exercise, Exercise therapy, Prostate cancer treatment

## Abstract

Prostate cancer (PCa) affects 1 in 8 men, but exercise therapy has been shown to be a very effective intervention not only to induce physiological benefits but to also reduce the side effects of cancer treatments typically administered during PCa. The COVID19 pandemic has restricted access to exercise clinics, a problem which always existed for people living in rural and remote areas. This caused many exercise physiologists and researchers to transition their clinic-based exercise to online, home-based exercise. We would like to propose that researchers and exercise physiologists should consider the use of elastic tubes in both research and the clinical management of PCa, when exercise programs are administered remotely, as their characteristics make them an ideal exercise equipment. In this article, the characteristics, considerations, and information on quantifying exercise dosage when using elastic tubes in remote exercise delivery are discussed.

## Clinic-based to home-based exercise medicine for prostate cancer

According to the American Cancer Society and Cancer Council Australia, 1 in 8 men in the USA and 1 in 6 men in Australia will be diagnosed with prostate cancer (PCa) during his lifetime. Exercise therapy has been shown to be a very effective intervention not only to induce physiological benefits but to also reduce the side effects of cancer treatments typically administered during PCa. Specifically, we have previously shown that resistance exercise training (RET), performed 2–3 times per week for 12 weeks, prevented muscle loss in PCa patients undergoing androgen-deprivation therapy, even when combined with radiotherapy [1]. Moreover, 12 weeks of RET enhances the expression of serum myokines with tumor-suppressive effects in PCa patients [2]. Therefore, RET is an important intervention that can both exercise tumor-suppressive effects and also reduce the side effects of PCa treatments.

In the past 2 years, the COVID19 pandemic rendered a lot of people around the world unable to access exercise facilities, including PCa patients from receiving exercise therapy in exercise physiology clinics. In addition, the same challenge always existed with PCa patients who lived in remote or rural areas with no access to exercise physiology clinics. In the past year, many exercise physiologists and researchers transitioned their clinic-based RET to online, home-based RET. For example, in a recent paper, Winters-Stone et al. reported that they are conducting a home-based RET trial in PCa patients to investigate if it results in a fall reduction, by providing the participants with free weights to use in their homes [3]. We would like to propose that researchers and exercise physiologists should instead consider the use of elastic tubes in both research and the clinical management of PCa, when exercise programs are administered remotely.

## The value of elastic tubes as a telehealth tool

**Fig. 1:**
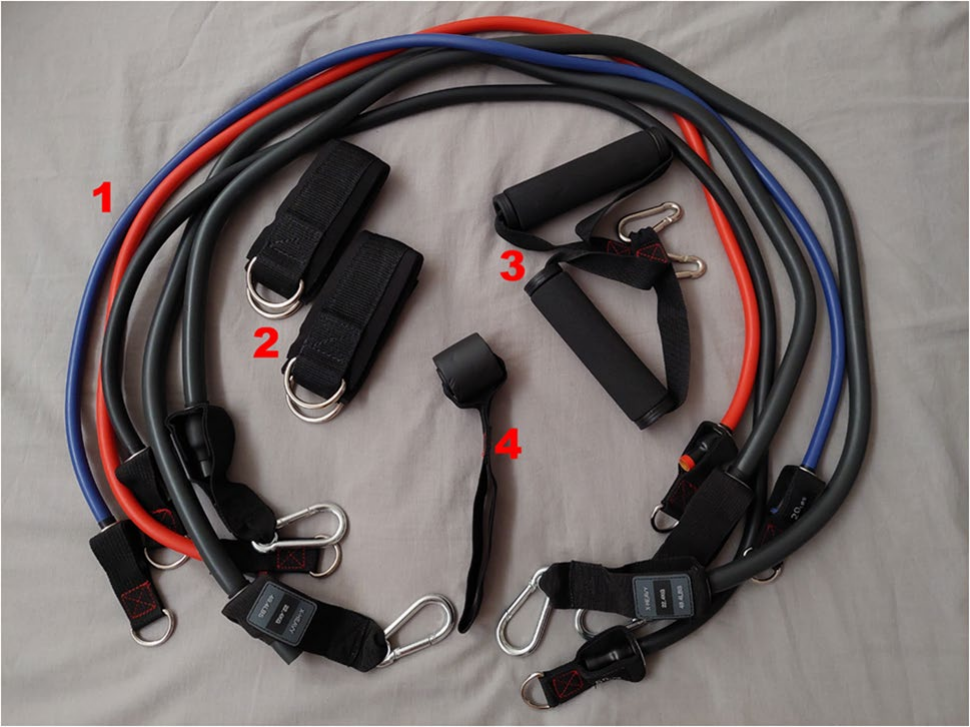
An elastic tube set. 1: The elastic tubes of different resistance levels, 2: ankle straps, 3: handles, and 4: door anchor. Each of these attachment points can be connected with multiple tubes at one time, to increase resistance as required

An elastic tube set is composed of different elastic tubes with metallic attachments at their ends, where grip handles or foot straps can be connected (Fig. 1). Commercial sets also contain door anchor points, to enable the performance of pulling exercises with two limbs. When multiple tubes are combined, they can result in loads of more than 70 kg, with multiple handles and foot straps to allow the execution of almost every exercise that is possible with gym machine equipment. Moreover, when considering the different characteristics of resistance exercise equipment, elastic tubes can be considered an ideal equipment for telehealth exercise therapy. Specifically, the equipment scores very high in terms of compactness, home usage, adjustability, portability, simplicity, multiplicity, noiselessness, light weightlessness, inexpensiveness, effectiveness, safety, durability, and not requiring supervision [4].

## Elastic tubes in prostate cancer telehealth

Exercise training using elastic tubes can effectively increase fat-free mass and muscle strength and decrease fat mass and frailty, to the same extent as free weights or machine equipment [5–9]. These findings are particularly important for PCa patients, which are typically over 65 years old, are sedentary, and in many cases frail [10]. In patients with PCa, frailty is associated with worse health-related quality of life [10], while higher muscle mass, and lower fat mass are associated with overall survival [11], and thus, exercise training with elastic tubes cannot only improve fitness, but can also result in crucial disease-specific benefits.

Transitioning from free weights or machine equipment can be confusing for the patients, as the resistance of the tubes is variable, and they cannot always perfectly approximate the load that would be explicitly indicated on free weights or machines. Regardless, one study in sedentary middle-aged women successfully used perceived exertion in the active muscles, increasing fat-free mass and strength to the same extent as weight machines [8]. Thus, we recommend using perceived exertion or effort, two concepts which can be easily understood even by patients unaccustomed to exercise terms and quantification of exercise dosage. PCa patients can be provided with elastic tubes, either for clinical practice or research, along with a guidebook, either printed or online, demonstrating the correct usage of the equipment, the available exercises, and a scale of perceived effort. In addition, the exercise routine can also be supplemented with bodyweight exercises.

Quantifying exercise dosage correctly is important to tolerability and adherence to exercise in people with cancer [12]. The patient can be prescribed specific sets and repetitions, and asked to attach different tubes for each exercise, to achieve a target perceived effort. The patient could then write down the total load from the combination of the tubes used, so that the dosage can be quantified. The length of the band greatly affects the elastic force exerted; therefore, the patient should be told to keep their distance from the attachment point constant. Moreover, the patient can also report if there were any myoskeletal pains, and the exercise program can be modified accordingly.

The field of telehealth exercise medicine for PCa is still emerging, and there is a lot of potential to improve the delivery of exercise therapy to PCa patients who have limited access to exercise physiology services. We urge researchers and exercise physiologists to consider the use of elastic tubes in both research and the clinical management of PCa, as an inexpensive, portable, safe, and effective exercise equipment.

## Data Availability

Microsoft Word
